# Quantifying Phytogeographical Regions of Australia Using Geospatial Turnover in Species Composition

**DOI:** 10.1371/journal.pone.0092558

**Published:** 2014-03-21

**Authors:** Carlos E. González-Orozco, Malte C. Ebach, Shawn Laffan, Andrew H. Thornhill, Nunzio J. Knerr, Alexander N. Schmidt-Lebuhn, Christine C. Cargill, Mark Clements, Nathalie S. Nagalingum, Brent D. Mishler, Joseph T. Miller

**Affiliations:** 1 Centre for Australian National Biodiversity Research, Commonwealth Scientific and Industrial Research Organisation, Plant Industry, Canberra, Australian Capital Territory, Australia; 2 School of Biological, Earth and Environmental Sciences, University of New South Wales, Sydney, New South Wales, Australia; 3 National Herbarium of New South Wales, Botanic Gardens Trust, Sydney, New South Wales, Australia; 4 University and Jepson Herbaria, Department of Integrative Biology, University of California, Berkeley, Berkeley, California, United States of America; 5 Australian Tropical Herbarium, James Cook University, Cairns, Queensland, Australia; State Natural History Museum, Germany

## Abstract

The largest digitized dataset of land plant distributions in Australia assembled to date (750,741 georeferenced herbarium records; 6,043 species) was used to partition the Australian continent into phytogeographical regions. We used a set of six widely distributed vascular plant groups and three non-vascular plant groups which together occur in a variety of landscapes/habitats across Australia. Phytogeographical regions were identified using quantitative analyses of species turnover, the rate of change in species composition between sites, calculated as Simpson's beta. We propose six major phytogeographical regions for Australia: Northern, Northern Desert, Eremaean, Eastern Queensland, Euronotian and South-Western. Our new phytogeographical regions show a spatial agreement of 65% with respect to previously defined phytogeographical regions of Australia. We also confirm that these new regions are in general agreement with the biomes of Australia and other contemporary biogeographical classifications. To assess the meaningfulness of the proposed phytogeographical regions, we evaluated how they relate to broad scale environmental gradients. Physiographic factors such as geology do not have a strong correspondence with our proposed regions. Instead, we identified climate as the main environmental driver. The use of an unprecedentedly large dataset of multiple plant groups, coupled with an explicit quantitative analysis, makes this study novel and allows an improved historical bioregionalization scheme for Australian plants. Our analyses show that: (1) there is considerable overlap between our results and older biogeographic classifications; (2) phytogeographical regions based on species turnover can be a powerful tool to further partition the landscape into meaningful units; (3) further studies using phylogenetic turnover metrics are needed to test the taxonomic areas.

## Introduction

The definition of biogeographical regions (also referred to as bioregions) is fundamental for understanding the distribution of biodiversity [1]. Bioregionalizations are important because they allow us to classify organisms into fundamental geographic units, at different scales, to be used to establish global conservation agreements and to make diversity assessments [2], [3], [4], [5]. The characteristics and terms used to define areas in biogeography are not always used consistently (Table 1). For example, biomes are defined by both the climate and the types of organisms that have adapted to it and floristic zones are defined only by the types of vegetation they contain. However, they are sometimes used interchangeably. Bioregions (phytogeographical and zoogeographical regions) are defined on the distributions of specific taxonomic groups, and therefore are simpler to understand and to use comparatively.

**Table 1:** Glossary of terms.

Term name	Description
**Area**	The region of distribution of any taxonomic unit (species, genus, family) of the plant (or animal) world (Wulff 1950: 25) [51]. An endemic area is the geographical area to which a taxon or biota is understood to be native. (Parenti and Ebach 2009: 253) [50].
**Biome**	Bioclimatic Zone. The geographical area defined by climate and the types of organisms that have adapted to it (e.g., mesic, arid).
**Biota**	A group of taxa (organisms), the combined distribution of which occupies a common set of geographical limits. (Parenti and Ebach 2009: 252) [50].
**Biotic Area**	The geographical area inhabited by a biota. Limits of taxon distribution specify limits of the area (Parenti and Ebach 2009: 251) [50].
**Biogeographical Region**	Bioregion or phytogeographical and zoological regions. The geographical area based on the distributions of specific taxonomic groups (e.g., plant or animal taxa).
**Vegetative (Floristic) Zone**	The geographical area defined by a particular type of vegetation (e.g., savannah, tundra, Mulga Scrub).

The terms, regions, areas, and vegetation are often used inter-changeably, however, they do have specific meanings that we use herein with the following definitions.

The history of Australian bioregionalization spans 190 years [6] and may be divided into the colonial, post-federation, ecogeographical and systematic periods. The first attempt at a bioregionalization classification of Australia was by Ferdinand von Mueller in 1858, using vegetation types rather than taxic distributions [7]. In contrast, the naturalist Ralph Tate in 1889 produced the first bioregionalization using a combination of taxic distributions and climate, coining the terms Eremaean and Euronotian, both of which are still in use [8] and are also used here. In 1933, during the Post-federation period, the zoogeographer G.E. Nicholls presented the first combined regionalization of Australian terrestrial flora and fauna [9]. Rather than adopt a combined bioregionalization, however, many Australian plant and animal geographers chose instead to keep animal and plant distributions separate. Most important was Nancy Burbidge, who by the Ecogeographical Period developed her own regionalization consisting of areas that were largely based on Tate's work [8] as well as her expert knowledge of flora distributions. Burbidge's classification of Australia flora used the concept of interzones, that is, areas of overlap (including the MacPherson-Macleay overlap zone), along with three floristic zones (Temperate, Tropical and Eremaean) [10]. Possibly the last continental bioregionalization of Australia's flora was made by Dutch botanist Henk Doing in 1970, who created the first hierarchical classification of Australian flora according to vegetation type, communities and climate [11].

Other studies attempted to regionalize Australia through using numerical, evidence-based data, such as Barlow's 33 botanical regions derived from herbarium specimens [12]. By the 1980s regionalization was done at the regional rather than continental level and many classifications ignored biotic areas in favour of vegetative or climatic zones (i.e., biomes) [13]. However, by the time of the Systematic Period, particularly in the early 1990s, there was a resurgence of biotic area classification [14], [15], [16], [17]. Of these new classifications, very few were in agreement, leaving phytogeography with more areas and area names than ever before [18]. In summary, there has to date been an accumulation of, often conflicting, area classifications, none of which were quantitatively produced or assessed. Modern advances in the development of large databases of georeferenced specimen observations, allied with concurrent improvements in spatial analysis tools, means that it is now possible to quantitatively define and assess biotic regions [19], [20].

A key concept to define biogeographical regions (herein phytogeographical regions) is species turnover, which is the rate of change in species composition between sites [21]. Species turnover has been used to generate classifications of bioregions [19], [20], [22], [23], [24], [17] and there have been some studies, for example, in sub-Saharan regions of Africa, where multiple taxa were used to successfully partition the continent into phytogeographical regions [25]. In other cases, species turnover and multivariate statistical methods effectively diagnosed bioregions on different biological groups [26], [27], [28], [29], [30]. However, no large dataset of geo-referenced taxa has been used to identify phytogeographical regions of plants across an entire continent such as Australia. Moreover, there has been no quantitative attempt to test existing phytogeographical regions that have been in use since the late 19^th^ century [8].

The method we apply to quantifying phytogeographical regions is similar to recent zoogeographical studies [1], [24]. Limited access to large nationally digitized spatial datasets is a likely reason why large studies do not exist on this topic. Australia is an exception because of the existence of Australia's Virtual Herbarium AVH [31], which has digitized most specimens housed in herbaria around Australia (http://www.avh.ala.org.au/). This amalgamated database of herbarium records for an entire continent makes it possible to investigate large scale patterns of plant distributions. In this paper we used quantitative methods to prepare a phytogeographical classification for the entire Australian continent using species turnover of nine major plant groups (Table 1) and to test the validity of an existing classification of Australia's three regions and 18 sub-regions. Our dataset contains representatives from diverse land plant groups (bryophytes, ferns, and angiosperms).

By first developing a species turnover-based phytogeographical classification, using taxonomic groups instead of climate, we are then able to test how the environmental patterns, such as of climate and soil types [19], [20], fit the observed phytogeographical regions.

The aims of this study were to:

1. Quantify and map the plant regions of Australia through spatial analyses of modern databases of georeferenced specimen data and compare these with the current phytogeographical regionalization.
2. Identify what the major environmental drivers of species turnover are for each of these phytogeographical regions.
3. Test the validity of previously proposed regions and sub-regions from the last 190 years, and propose an improved classification of the phytogeographical regions of Australia.

## Methods

### Spatial dataset

Table 2 summarizes the taxa and number of occurrence records examined in this study. A total of 802,273 records were downloaded from *Australia's Virtual Herbarium* (AVH) [31]. This dataset does not contain absence records, of course. Collections were mostly curated to the accepted taxonomy of the *Australian Plant Census* [32]. We did not consider any infra-specific taxa in the analysis. For each species, spatial errors were removed using ArcGIS 9.2 [33]. The spelling and consistency of scientific names across taxa were corrected using Google Refine. Spatial outliers and all records without a geographic location were deleted. Records that fell in the ocean or outside continental Australia were excluded. After the correction process, 750,741 records remained for use in our final analyses. The geographic coordinates of each record were projected into an Albers equal area conic conformal coordinate system to avoid the latitudinal biases of geographic coordinate systems. The records were imported and aggregated to 100 km×100 km grid cells (870 in total) using Biodiverse 0.18 [34] (http://purl.org/biodiverse). We calculated the ratio of species records to number of samples per grid cell to measure redundancy, as an indicator of sample coverage [35]. We found that 70% of the analyzed grid cells had a good level (60%) of species record redundancy within each grid cell (see Appendix S1).

**Table 2:** The plant groups used in this study, number of occurrence points, and the number of species per group, with the totals.

Taxon name	Number of records	Number of species
*Acacia*	165,518	1,020
Asteraceae	105,692	823
Eucalypts (*Angophora, Corymbia and Eucalyptus*)	202,736	791
Ferns	58,774	356
Hornworts	370	13
Liverworts	16,502	735
Melaleuca	41,092	282
Mosses	79,210	835
Orchids	80,847	1,188
**TOTAL**	**750,741**	**6,043**

### Taxonomic dataset

The dataset included Australian representatives from a diverse set of major terrestrial plant groups (bryophytes, ferns and several large angiosperm genera and families) that represent a wide geographic distribution across Australia [53], [54]. For example, *Acacia* and eucalypts are the most abundant canopy and sub-canopy woody plants in Australia [36], [37], [53]. One of the problems when gathering a large dataset is the reliability of the taxonomic identification. The more taxa included, the more challenging it becomes to achieve high taxonomic and spatial reliability of herbarium data. The strategy we applied was to utilize as many taxa as possible that combine a strong taxonomic tradition and wide geographical ranges. Despite having experts on each group involved in the cleaning process checking for taxonomic and spatial errors in the sampled groups, we expect there to remain some low degree of taxonomic uncertainty.

### Spatial analysis: bioregionalization

All spatial analyses were conducted using Biodiverse [34]. A matrix of species turnover was generated for all pair-wise combinations of grid cells (757,770 pairs). Simpson's beta (β_sim_) was used as the turnover measure because it corrects for species richness differences between sites (Equation 1). 
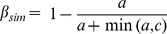
(Eq1.)


Where a refers to the number of species common to cells i and j, b is the number found in cell i but not cell j, and c is the number found in cell j but not cell i. A low β_sim_ value indicates that many taxa are shared between two grid cells (low dissimilarity) and a high β_sim_ means a small number of shared taxa (high dissimilarity).

An agglomerative cluster analysis of the βsim turnover matrix was used to generate a WPGMA hierarchical cluster diagram in Biodiverse. Current literature suggests that dissimilarity clustering algorithms are exposed to topology biases [55]. In order to reduce biases in our analyses, the cluster analysis included a tie-breaker condition [18], [19] such that, when more than one pair of sub-clusters had the same turnover score, the pair which had the highest Corrected Weighted Endemism (CWE) score was selected for merging. This approach guarantees the same cluster result would be generated each time the model is run, as well as increasing the endemicity of the resultant clusters. We identified the phytogeographical regions from the clusters based on two criteria: (1) a phytogeographical region is preferably represented by a group of contiguous, or near-contiguous, grid cells; (2) each cluster that represents a phytogeographical region needs to be clearly separated from its children (immediate descendent nodes) or parent (immediate ancestral node) in the dendrogram.

### Spatial overlap analysis

Our phytogeographical regions were visually compared with several previous bioregionalizations of Australia. We also conducted a formalized comparison of the degree of overlap between our phytogeographical regions and the terrestrial phytogeographical sub-regions proposed in the Australian Bioregionalization Atlas (ABA). This comparison used only the ABA because it is the existing classification that best reflects a historical viewpoint of geographical regionalizations of flora for Australia. Both classifications were converted to raster format with a resolution of 100×100 km. Because we aimed to identify phytogeographical regions at the continental scale, a coarse grid cell size was applied. The effect of changes in grid cell size on the bioregions was explored in prior studies with eucalypts across Australia [19]. In that study we found no significant implications on the identification of the major regions but some changes in the delimitation of the regions boundaries occurred. The ABA sub-regions were then overlaid on our phytogeographical regions using ArcMap 9.2 [33]. The overall spatial agreement between both classification schemes (which differ in the number of classes and their extent) was calculated by the count of ABA sub-region cells that overlapped with only one of our regions and then divided by the total number of cells in all ABA sub-regions. This value was summed up and expressed as a percentage of overlap.

### Environmental correlates

Eleven environmental variables were used in this study (Table 3). A correlation matrix available on the spatial portal of the Atlas of Living Australia was used to select variables which represented different environmental traits and demonstrated minimal correlation. The spatial resolution of the layers was 1 km (approximately 0.01 degrees). The environmental layers were re-projected into the same Alber's conic conformal coordinate system as the species data using the R software [38] and aggregated to 100 km×100 km grid cells using Biodiverse. The environmental variables were developed using ANUCLIM [39], [40]. We also included four layers related to soils and topography, sourced from the National Land & Water Resources Audit [41]. The mean value for each environmental variable within each 100 km×100 km grid cell was calculated using Biodiverse.

**Table 3:** Environmental variables used in our analyses.

Environmental variable	Description
Annual precipitation	Monthly precipitation estimates (mm)
Annual mean temperature	The mean of the week's maximum and minimum temperature (°C)
Annual mean radiation	The mean of all the weekly radiation estimates (Mj/m^2^/day)
Precipitation of coldest quarter	Total precipitation over the coldest period of the year
Radiation seasonality	Standard deviation of the weekly radiation estimates expressed as a percentage of the annual mean (Mj/m^2^/day)
Precipitation seasonality	Standard deviation of the weekly precipitation estimates expressed as a percentage of the annual mean (mm)
Temperature seasonality	Standard deviation of the weekly mean temperatures estimates expressed as a percentage of the annual mean (°C)
Ridge top flatness	Metric of the topographic flatness derived from a surface of 9 second grid cells (dimensionless)
Rock grain size	Lithological property of the bedrocks related to the mean grain size (0–10 units)
Sand	Content of sand on the top 30 cm of soil layer estimated from soil maps at a resolution of 1 km (%)
Clay	Content of clay on the top 30 cm of soil layer estimated from soil maps at a resolution of 1 km (%)

Relative environmental turnover (RET) aims to identify phytogeographical regions based on species turnover and investigate their environmental correlates. It has been shown to be a useful method to partition the continent into meaningful phytogeographic regions in *Acacia* and eucalypts [18], [19]. RET was derived from a framework to delineate biogeographic regions initially proposed by Kreft & Jetz [23]. Previous studies used the term environmental turnover to explore rates of change of dissimilarity in vertebrates and their relationship to environment depending on the geographic distance [24]. RET is different from previous approaches because it is not geographic distance based, but instead combines grid cell analyses with ordinations. RET consist of two parts, one is an ordination and the second is a gridded analysis. Here, we only used the gridded component of RET. The key question addressed is what are the main environmental differences among the phytogeographic regions that were inferred from species turnover?

The gridded approach consists of three steps. First, summary statistics were calculated for 100 km×100 km grid cells of the eleven environmental variables in Biodiverse. Then, each of the grid cells of the environmental variables were spatially linked to each of the grid cells corresponding to the phytogeographic regions. Finally, the association between the phytogeographical regions and the environmental variables were calculated using Getis-Ord Gi* hotspot statistic spatial statistics in Biodiverse. We used the Getis-Ord Gi* hotspot statistic to assess if the environmental values within the clusters (phytogeographic regions) were significantly different from those for the country as a whole [42], [43]. The Gi* statistic is expressed as a z-score indicating the degree to which the values of a subset of grid cells, in this case the cells comprising a cluster, are greater or less than the mean of the dataset. Those clusters with Gi* values greater than 2 or less than −2 represent sets of cells that have environmental values significantly different from expected (α<0.05).

## Results

### Phytogeographical regions

**Figure 1:**
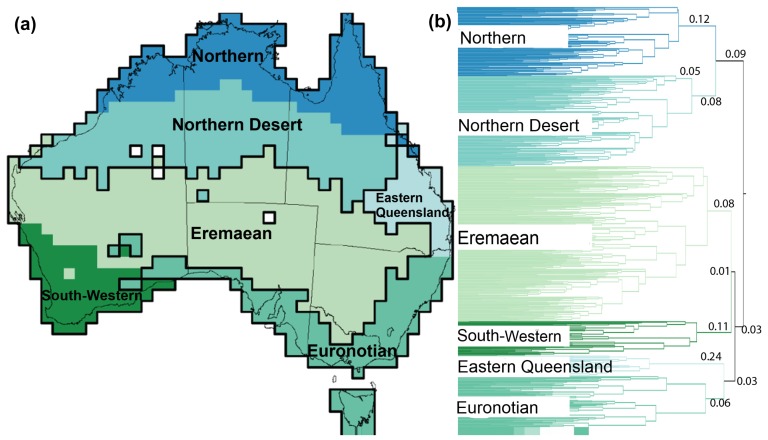
Phytogeographical regions of Australian terrestrial flora (a) as defined by the corresponding dendrogram (b). The colors of the regions in the map correspond to those used to plot the dendrogram. The dendrogram is a representation of the spatial relationship of dissimilarities in species composition among regions.

We found and propose six phytogeographical regions: Northern Region (Euronotian, Monsoonal Tropics and Monsoon *sensu* [57], [18], [19]), Northern Desert Region (Eremaean North *sensu* [18], [19], Eremaean (Eremaean South *sensu* [18], [19]), Eastern Queensland (South-eastern Temperate and southeast *sensu* [14], [18], [19] and South-Western (Southwest *sensu* [8]) (see Fig 1). The names of the proposed regions are aligned to correspond to the Australian Bioregionalisation Atlas [17].

**Figure 2:**
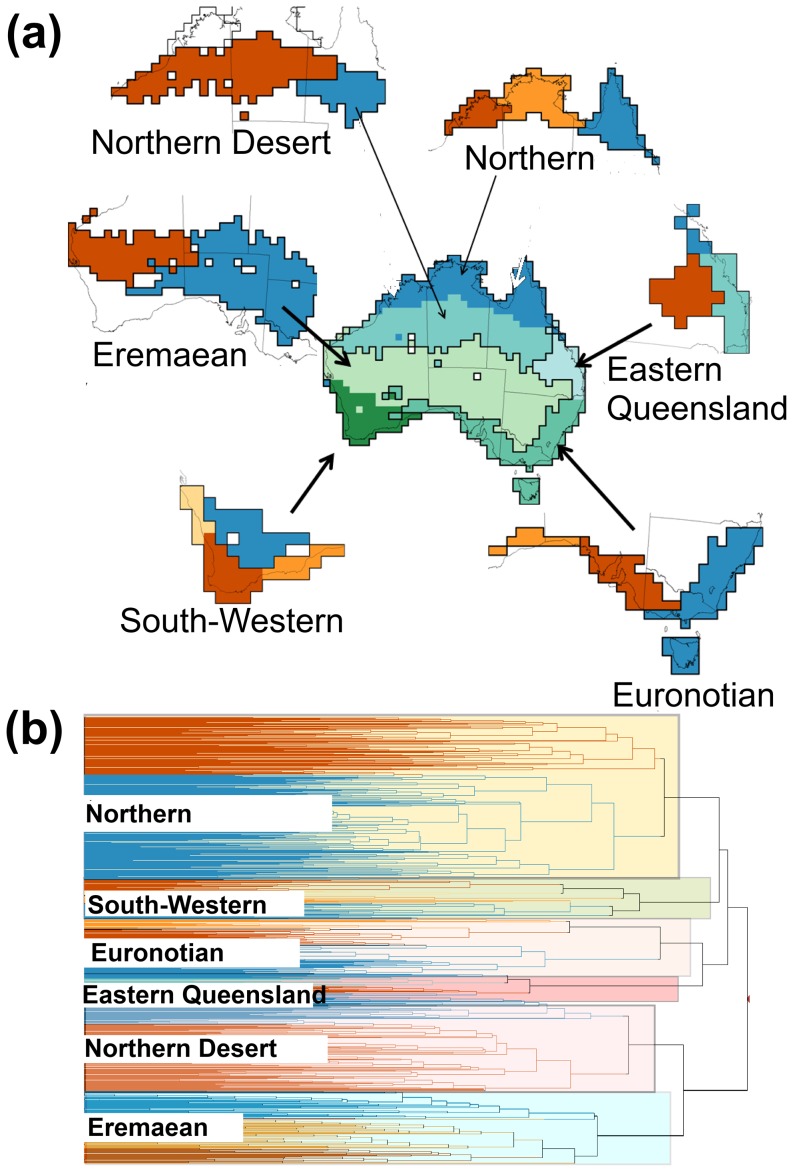
Phytogeographical regions and new subregions proposed for Australia (a), and their corresponding dendrogram (b). Note that the colors of the dendrogram clusters correspond to the colors of the subregions. Shaded colors indicate relationships: light blue and dark blue cluster together before clustering with brown colours.

Overall, the spatial arrangement of the phytogeographical regions follows a distinctive north to south pattern, with an east to west pattern at the sub-regional level. The phytogeographical regions are nested in geographically related pairs. The first split is of the Northern and Northern Desert regions (branch length (bl) of 0.09; dendrogram in Fig 1) from the other four regions. The Northern is on a long branch (bl = 0.12) and readily subdivided east (bl = 0.09) to west (bl = 0.03; Fig 2). The region with the highest species similarity to the Northern is the Northern Desert Region (bl = 0.08; Fig 1). This phytogeographical region also has a clear east (bl = 0.05) to west subdivision (bl = 0.08; Fig 2).

The other major cluster has a short branch (bl = 0.03). The largest clustering of grid cells is the Eremaean phytogeographical region which shares a very short branch (bl = 0.01) with the South-Western phytogeographical region (Fig 1). The Eremaean is on a long branch (0.08) and subdivides east (bl = 0.07) to west (bl = 0.04; Fig 2). The South-Western phytogeographical region is also on a long branch (bl = 0.11) and is subdivided into three coastal (bl = 0.02) subregions and an inland (bl = 0.04) subregion (Fig 2).

The Euronotian and Eastern-Queensland phytogeographical regions cluster together (bl = 0.03). The Eastern Queensland phytogeographical region (bl = 0.24) runs along the eastern coast of Queensland from the Wet Tropics to the New South Wales border and inland into south central Queensland (Fig. 1). This region subdivides into northern and southern coastal subregions (bl = 0.03) and an inland (bl = 0.07; Fig 2) subregion. The Euronotian phytogeographical region (bl = 0.06) has a strong east subregion (bl = 0.16) to central-west subregion (bl = 0.12) structure (Fig. 2).

### Environmental correlates of the phytogeographical regions

The environmental correlates of the six phytogeographical regions are shown in Table 4. The most extreme Gi* score was a precipitation trait for four of the six phytogeographical regions, while a temperature trait was the most extreme for the other two (see underlined values in Table 4). Species distribution and bioregions of *Acacia* and eucalypts in Australia are strongly influenced by annual precipitation and seasonal temperatures as well [18], [19].

**Table 4:** Gi* spatial statistics for the six phytogeographical regions of Australian flora. Bolded means statistically significant (α = 0.05).

Environmental variable	Northern (N = 141)	Northern Desert (N = 185)	Eremaean (N = 317)	Eastern Queensland (N = 43)	Euronotian (N = 114)	South-Western (N = 70)
Annual mean radiation	**3.37**	**9.04**	**4.91**	**−2.03**	**−14.90**	**−6.75**
Annual mean temperature	**12.69**	**11.66**	**−2.55**	−0.82	**−17.02**	**−8.45**
Annual mean precipitation	**17.69**	**−4.89**	**−15.45**	**5.01**	**6.86**	−1.78
Clay	**−2.28**	1.16	**2.05**	**3.85**	0.37	**−5.81**
Precipitation coldest quarter	**−7.31**	**−7.76**	**−5.51**	1.83	**16.26**	**9.71**
Precipitation seasonality	**17.38**	**13.23**	**−12.83**	−1.61	**−12.59**	**−3.84**
Radiation seasonality	**−14.83**	**−11.44**	**7.22**	**−2.44**	**15.81**	**6.86**
Ridge Top flatness	−1.90	**3.29**	**3.60**	**−3.16**	**−5.74**	0.89
Rock grain size	1.52	**−4.71**	−0.20	−0.89	−1.31	**7.70**
Sand	**3.31**	0.78	−2.26	**−2.79**	−1.84	**2.85**
Temperature seasonality	**−18.07**	0.87	**18.42**	-0.86	**−4.80**	**−2.77**

N =  number of grid cells per region. Underlined values are the most extreme scores for each region.

Precipitation and temperature seasonality are the environmental variables that better correlate with turnover of the Northern phytogeographical region (which could be termed the “monsoonal region” environmentally). The Eremaean phytogeographical regions are differentiated by seasonality traits in the Northern Desert Region and annual precipitation in the Eremaean, reflecting a possible Tropic of Capricorn division [10]. For the Euronotian phytogeographical region, the amount of solar radiation and precipitation during the coldest quarter of the year (winter) are the main environmental drivers. In the South-Western phytogeographical region precipitation in the coldest quarter, temperature, and landscape properties are the main drivers.

### Spatial comparison of the new phytogeographical regions to the ABA terrestrial sub-regions

**Figure 3:**
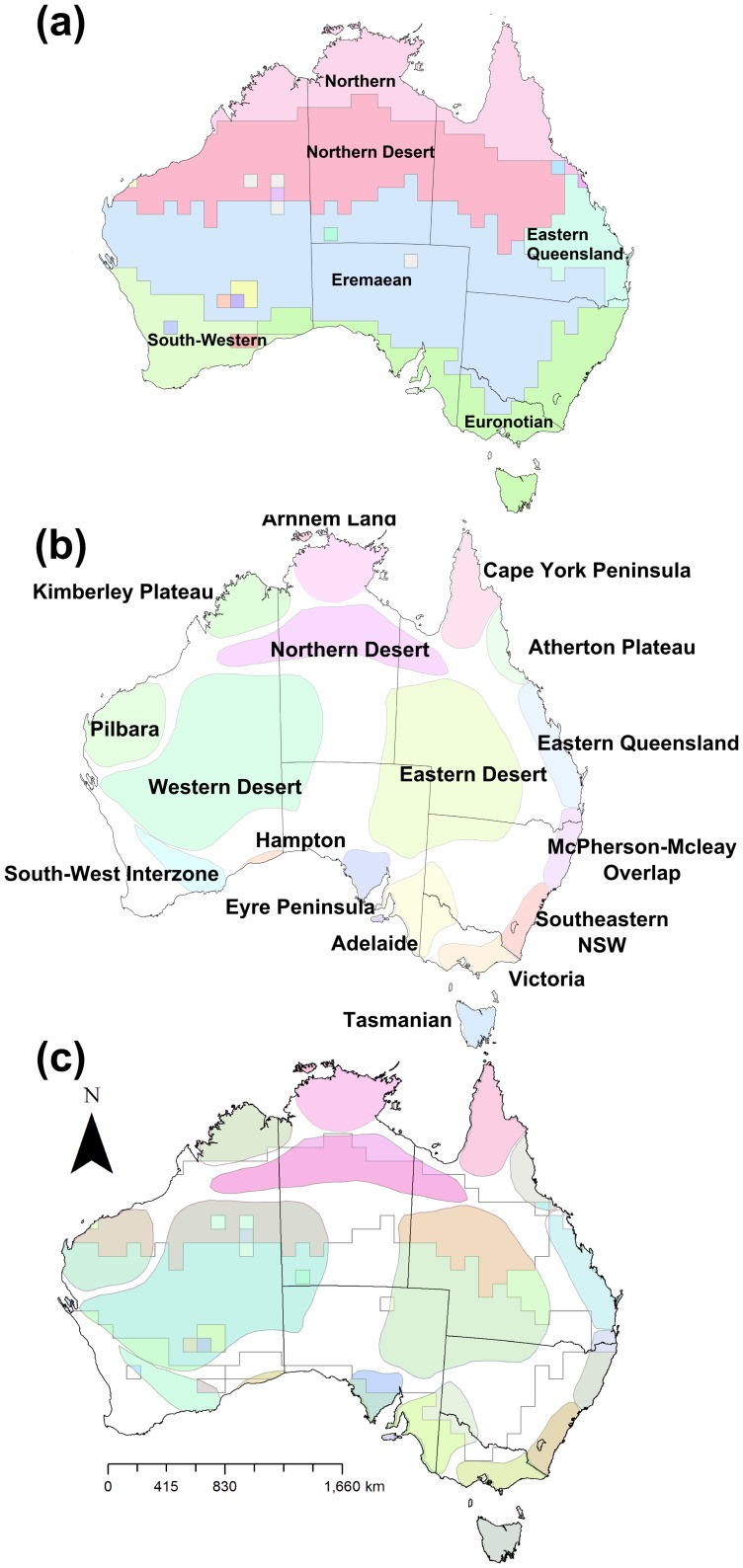
Spatial agreement between our six phytogeographical regions of Australian flora (a) and the terrestrial phytogeographical sub-regions of Australia (ABA) (b) [17]. Shown is the degree of spatial agreement of the ABA and our classification (c) and the percentage of overlap among each of our phytogeographical regions and the ABA sub-regions (d). Equivalent sub-regions from the ABA are noted below and as shown in Fig 2. The Northern Desert Region (red and blue: Northern Desert); Eremaean (red: Western Desert, blue: Eastern Desert); South-Western (blue & orange: Southwest Interzone); Euronotian (red: Eyre Peninsula and Adelaide [in part], blue: Victoria, Southeastern NSW, McPherson - Macleay Overlap [in part]); Eastern Queensland (blue: Atherton Tableland [in part], light blue: Eastern Queensland); Northern Region (red: Kimberley Plateau, orange: Arnhem Land, blue: Cape York Peninsula and Atherton Tableland in part).

Our phytogeographical regions resemble the nomenclature proposed by the terrestrial phytogeographical sub-regions of the ABA (Fig. 3c) [17] (Table 5). In numerical terms, the spatial agreement, between our phytogeographical regions (Fig 3a) and the ABA classification scheme is 65% (Fig 3c). This result represents a high level of agreement but there are still major gaps among many of the ABA subregions that we were able to fill using the species turnover approach.

**Table 5:** Area taxonomy overlaps between new areas and existing regions and sub-regions from the recently published Australian Bioregionalization Atlas (ABA) [17].

New Areas (this study)	ABA Area Taxonomy Regions	ABA Area Taxonomy Sub-regions
Northern Desert Region	Eremaean	Northern Desert
Eremaean	Eremaean	Western and Eastern Deserts
South-Western	Southwest Australia	Southwest Interzone
Euronotian		McPherson - Macleay Overlap (in part), Southeastern NSW, Victoria, Adelaide (in part), Eyre Peninsula (in part).
Northern Region	Euronotian	Kimberly Plateau, Arnhem Land, Cape York Peninsula, Atherton Tableland (in part)
Eastern Queensland	Euronotian	Atherton Tableland (in part), Eastern Queensland

Note that the new areas abut, while the ABA sub-regions are occasionally separated by undescribed areas (see gaps between regions in Figure 3b).

## Discussion

Our data suggest that the Northern region overlaps with the ABA Kimberly Plateau, Arnhem Land, Cape York and Atherton Plateau [in part] sub-regions and has a species composition more similar to the Northern Desert than to the more mesic phytogeographic area along the eastern coast of Australia. The Northern Desert phytogeographic region overlaps with some parts of the Northern, Eastern and Western Desert ABA sub-regions. The arid zone in our classification split into the Eremaean (including the southern parts of the Eastern Desert and western Desert ABA sub-regions) as well as South-Western region, which is considered one of the world diversity hotspots.

These results are similar to the proposed ABA phytogeographical regions and sub-regions that have been in use for over 120 years see [6], [7], [8], [9], [10], [11], [12], [13]. However, because they are based on a rigorous quantitative analysis of a large data set, our results should be used to revise the ABA area taxonomy and area boundaries as well as to extend sub-regions within the Eremaean and Euronotian regions, so that all areas abut. The current provisional area taxonomy within the ABA has few abutting sub-regions (see Figure 3b); our results can re-define these existing areas to create a more accurate area taxonomy for Australia's phytogeographical regions and sub-regions.

### Spatial comparison of the new phytogeographical regions to biomes

Our results strongly reflect the northern tropical summer and southern temperate winter rainfall gradients. Precipitation is a more significant environmental correlate in the northern half of the continent whereas high levels of solar radiation and cool temperatures are more important below the Tropic of Capricorn. However, annual precipitation is a predominant correlate of the coastal Queensland region where a tropical/sub-tropical transition zone, the Eastern Queensland phytogeographical regions, is created.

The north - south split between the Eremaean and Northern Desert Region roughly coincides with the Tropic of Capricorn and the summer-winter rainfall line (see Appendix S1 panel a). However, this split is not evident in the previously published biomes or bioregions of Australia (see Appendix S1 panels a-b-d) [10], [4], [44], [49]. These biome descriptions, which are defined by both climate and biota, identify a large arid Eremaean region that is not split north to south into two regions as was found in our analysis. The Eremaean “zone is crossed obliquely by the junction between the summer and winter rainfall systems but floristically the junction is not so strongly marked due to the presence of small ranges of low mountains, which appear to have acted as refugia” [10]. Our evidence suggests that the division line between Eremaean and Northern Desert regions might be related to the effect of the Tropic of Capricorn, which may have resulted from the palaeoclimatic shifts (warmer-cooler-warmer) during the last 65 Ma [58]. It was mentioned in a compilation of Australian phytogeography that “floristic composition from north to south is probably as closely related to temperature gradient and possibly also day length as to available rainfall” [10]. We also observed a west-east climatic division within the Eremaean and Northern Desert regions. Our analysis identifies the Eastern Queensland as a separate phytogeographical region. This region can be described climatically as an inter-zone defined by the summer-winter rainfall variation as previously noted in Burbidge's biomes in Australia [10].

### Spatial comparison of the new phytogeographical regions to other classifications

The comparison of our regions and sub-regions against geology [45], soils [46], and vegetation types [47] uncovered few congruent patterns (Appendix S1). The results align with the current distribution of major vegetation groups of Australia as cited by the National Vegetation Information Systems (NVIS) [47]. Geology and soils are treated as artificial units (e.g., Formations, Ferrosols etc.), rather than types of rock and soil (e.g., sandstones, sandy loams) and therefore are unlikely to overlap. However, general climatic maps correlate with our results. The six proposed floristic regions (see Appendix S1 panel c) closely agree with Köppen's macro-climatic map of Australia (Appendix S1 panel e; http://www.bom.gov.au/climate/environ/other/kpn_group.shtml) [27]. Regarding Köppen's classification, the tropical zone (see Appendix S1, dark green in panel 3e) maps precisely with the Northern region, the subtropical zone (see Appendix S1, light green in panel e) matches well with our Eastern-Queensland region and the temperate climate group (see Appendix S1, blue in panel e) fit well with our Euronotian region. The main inconsistency is with the desert and grassland groups (see Appendix S1 orange and yellow in panel e) where a split into grassland that covers semi-arid areas is conspicuous, although these grassland areas roughly agree with the eastern subregions of the Northern Desert and Eremaean regions.

### Utilization of phytogeographical classifications

Our results support some previous biotic [57] and climatic classifications of Australia [10] but also disagree in some cases [59], and thus add new information to the biogeographical literature. For example, our results suggest for the first time that the flora of arid Australia (Eremaean Region of the ABA) can be divided into distinct phytogeographic regions, first along a north to south gradient and then along an east to west gradient, in contrast to some proposed biogeographic faunal patterns [48]. We show that a unified method for quantifying species turnover can be used to successfully partition a continent into geographically meaningful regions using a broad sample of plant groups. This analysis also demonstrates that biogeographical regionalisation does not have to be convoluted and complex. With fewer factors involved, patterns are easier to explain. For example, the strong evidence of the relationship of sub-regions of species turnover with climatic variables suggests that species assemblages across Australia have responded to changes in weather systems across the continent.

The phytogeographical regions presented here are defined using species turnover and thus relate to taxonomic diversity in the groups studied. Here, the taxonomic groups contain a combination of recent (*Acacia*) and older clades (the bryophyte groups). It is probable that the recently diverged clades are driving the patterns identified because they comprise a large proportion of the species sampled. However, the older bryophytic and pteridophytic clades do not have the same broad continental distributions of the younger clades studied here, which may reflect recent distributional patterns that might not be shared with these older clades. If dominated by the distributions of recently derived species, our results likely will match modern climatic zones, while older species might reflect geological features, tectonic patterns or older palaeo-climatic zones.

Given this, we highlight the importance of generating regionalizations based on large, multi-taxon datasets. Furthermore, basing floristic regions only on species turnover misses out on the full depth of phylogenetic information available. Future studies should compare these results with patterns of spatial similarity generated using measures of phylogenetic turnover [52], to obtain a better picture of the historical relationships among areas within Australia. Understanding the adaptive changes in morphology and physiology that accompanied biome shifts will enable a broad understanding of the adaptive history of organisms and its potential for adaptation in the face of human induced climate change [56].

## Supporting Information

Appendix S1 Comparison of our six phytogeographical regions of Australian flora (c) against major biogeographical classifications of Australia. Burbidges biomes [10] (a), Crisp et al biomes [49] (b), IBRA regions [4] (d) and Köppen's macro-climatic map of Australia (e). There is permission to re-print maps on panels A and B, and labels in panels D and E indicate the original publisher (official permission not required because is public material).
(TIF)
