# Supplementary figures and images for: Quantifying Phytogeographical Regions of Australia Using Geospatial Turnover in Species Composition
Source: PLoS One. 2014 Mar 21;9(3):e92558. doi: 10.1371/journal.pone.0092558 (PMC3962426; doi:10.1371/journal.pone.0092558)

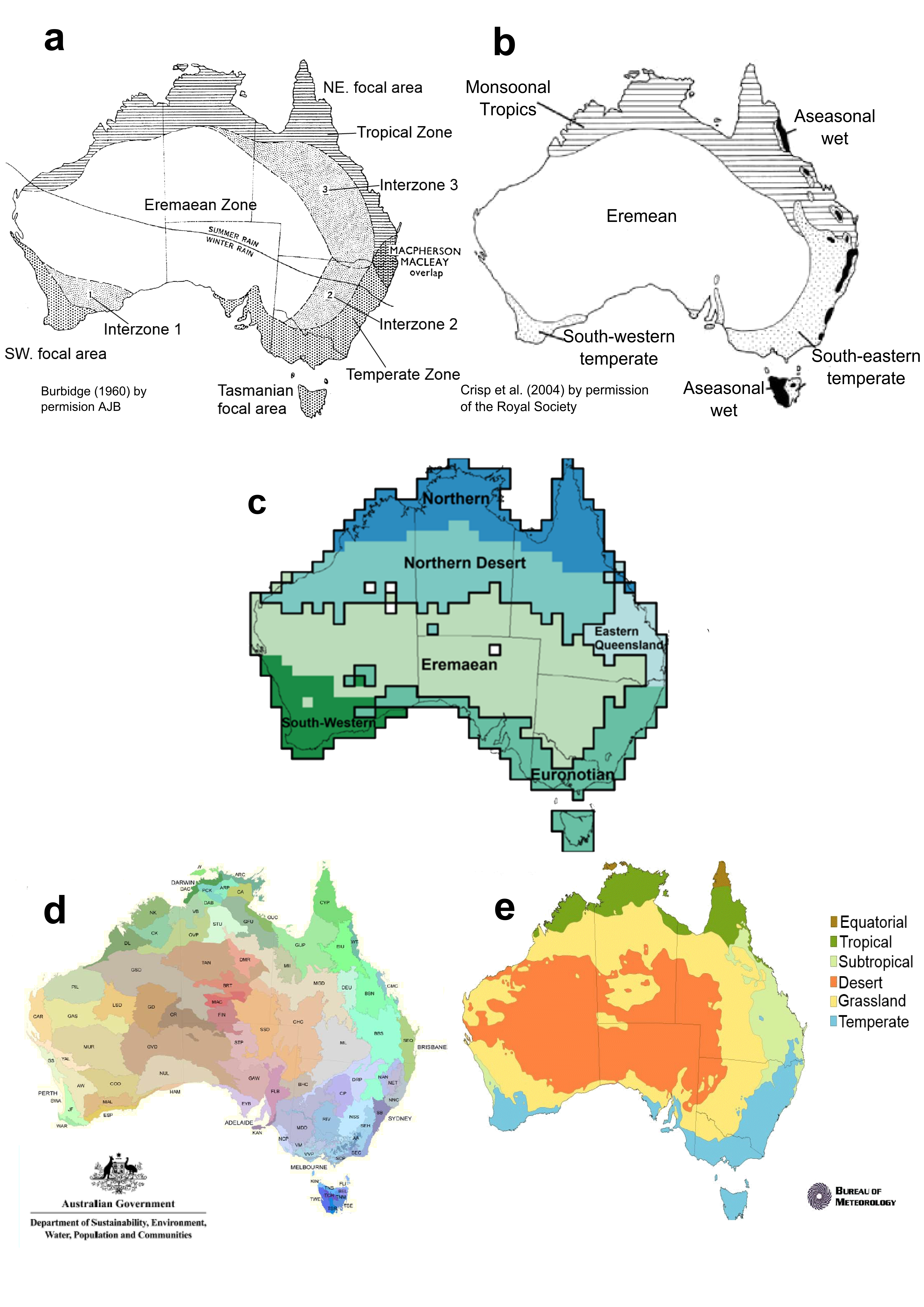

Supplement: Appendix S1 — Comparison of our six phytogeographical regions of Australian flora (c) against major biogeographical classifications of Australia. Burbidges biomes [10] (a), Crisp et al biomes [49] (b), IBRA regions [4] (d) and Köppen's macro-climatic map of Australia (e). There is permission to re-print maps on panels A and B, and labels in panels D and E indicate the original publisher (official permission not required because is public material). (TIF) [file pone.0092558.s001.tif]
